# Supplementary material for: A machine learning approach to identifying important features for achieving step thresholds in individuals with chronic stroke
Source: PLoS One. 2022 Jun 17;17(6):e0270105. doi: 10.1371/journal.pone.0270105 (PMC9205506; doi:10.1371/journal.pone.0270105)
Supplement: S1 File — (DOCX) [file pone.0270105.s003.docx]

A machine learning approach to identifying important features for achieving step thresholds in individuals with chronic stroke: Supplementary material

**Machine learning algorithms**

This section of the supplement is intended to provide a brief overview of the machine learning algorithms used in this study for readers unfamiliar with them. The three machine learning (ML) algorithms used in this analysis were Logistic Regression (LR), Support Vector Machine (SVM) with a radial basis function (RBF) kernel, and Random Forest (RF) classifier. These were chosen for this analysis because they are all commonly used supervised ML algorithms that are mathematically distinct from one another. A supervised classification algorithm is one that is trained by being provided with both data (X) and labels (Y), similar to a flash card. The way that supervised classification algorithms generally work is that they use the training data and labels to construct a function, f(x), to make predictions about which class other points in the same population belong to. Here, each point $x=(x^{1}, x^{2}, \ldots, x^{m})$ where $x^{k}$ is the value of x that corresponds with the k^th^ feature. This is done by solving an optimization problem to find the best parameters for f(x) based on what quantity is being optimized. The form of the predictor f(x) and the optimization process varies across ML algorithms.

In the case of LR with binomial class labels, as we have in our analysis, the general idea is that, based on our sample data, X, and labels, Y, we want to model the probability that Y=1, given X takes some value x, i.e.

$$p\left( x \right)=P\left[ Y=1 | X=x \right]$$

Then we also have that the probability that Y = 0 given X = x is 1- p(x). Now, to model this probability, p(x), LR uses the logistic function which takes values in (-∞, ∞) and maps them to the interval [0,1] (the values of the probabilities). During this process, we want to find some set of parameters, β (the vector of beta weights), that fit the data in some optimal way where the logistic function to model this probability is:

$$p\left( x,\beta\right)= \frac{e^{x^{T}\beta}}{1+e^{x^{T}\beta}}$$

Given our training data has n observations, the goal of LR is find the values of β that maximize the log-likelihood function:

$$LL\left( \beta\right)=ln\left( \prod_{i = 1}^{n} {p\left( x_{i},\beta\right)}^{y_{i}}\left( 1-p\left( x_{i},\beta\right) \right)^{{1-y}_{i}} \right)$$

$$= \sum_{i=1}^{n} y_{i}x_{i}^{T}\beta- ln\left( 1 + e^{x_{i}^{T}\beta} \right)$$

One important thing to note is that of the three models used in our analysis, LR is the most closely related to a linear model in the sense that the term $x^{T}\beta$ is a linear combination of the values of the vector x with the beta weights. This means that LR is only able to detect linear relationships between the features, where non-linear ones might exist.

For SVM, rather than computing the probability that a given observation belongs to some class, the goal is to establish boundaries with an allotted margin of error in the space the data comes from. These boundaries, which are defined by linear hyperplanes, separate the space the data comes from into regions. Then, a point would be classified depending on which of these regions the point lies in. In general, if our data set consists of m variables, then our data points live in an m-dimensional space. A hyperplane in an m-dimensional space would be defined by:

$$\beta_{0}+\beta_{1}x^{1}+\ldots+ \beta_{m}x^{m}=0$$

Which, in vector form, would be:

$$\beta_{0}+ \beta^{T}x=0$$

Where x is a single data point, $\beta_{0}$ is the bias term, and *β* is the vector of beta weights. Notice that, similar to LR, the linear hyperplane in the m-dimensional space is only able to use linear relationships between the variables, but it is very possible that the data is not linearly separable. This is where one of the benefits of SVM, the use of a kernel, comes into play. A kernel, such as RBF, allows for additional “new” variables to be constructed using non-linear combinations of the existing variables. This allows the data to be projected into higher, m+d dimensional space, where d is the number of “new” variables, in which the data may now be separable by a linear hyperplane in this new, higher dimensional space. The way this would change the formulation of the boundary would be:

$$\beta_{0}+ \beta^{T}\phi\left( x \right)=0$$

where $\phi$ is an unknown function of $x$ that is related to the transformation into higher dimensional space. The term $\phi\left( x \right)$ is what would be the non-linear part of the definition of the hyperplane in $m$-dimentional space.

The RBF kernel, which is defined by:

$${K\left( x_{i} , x_{j} \right)= e}^{{-\gamma(x_{i}-x_{j})}^{2}}$$

allows the data to be projected into an infinite dimensional space, but rather than ever actually making the physical transformation, the kernel functions allow for these higher dimensional relationships between the points to be computed because the kernel function acts as a dot product in that space. In this way, the RBF kernel can be thought of as a measure of how “close” two observations $x_{i}$and $x_{j}$ are in infinite dimensional space. What makes SVM so robust is not only that it can use a kernel but also that, when establishing this boundary, there are allowances for misclassification of training data to handle noise. The optimization problem then becomes maximizing the distance between the points and the hyperplane while trying to minimize misclassification error:

$$\max_{\beta_{0}, \beta, M, \varepsilon_{1},\ldots, \varepsilon_{n}} M$$

Subject to the constraints:

$$\left\| \beta\right\|=1$$

$$\left( 2y_{i}-1 \right)\left( \beta_{0}+ \beta^{T}\phi\left( x_{i} \right) \right)\geq M\left( 1-\varepsilon_{i} \right)$$

$$\sum_{i=1}^{n} \varepsilon_{i} \leq C$$

Where $M is the size of the margin,$ $\left( 2y_{i}-1 \right)\left( \beta_{0}+ \beta^{T}\phi\left( x_{i} \right) \right)$ is the distance from the observation $x_{i}$ to the hyperplane in the higher dimensional space, and C and $\varepsilon_{i}$ ≥ 0 are the total error tolerance and the “slack” allowed for each observation, $x_{i}$ , respectively. This optimization problem can be solved using a Lagrange multiplier, which eliminates the dependency on the unknown function $\phi\left( x \right)$. The end result is a potentially non-linear boundary that is as far away as possible from most points in each class, thus creating a robust classifier in the physical feature space.

Finally, RF is what is referred to as an “ensemble” because it is much more a collection of individual models, rather than a single model on its own. Simply put, a random forest is exactly what it sounds like: it is a collection (or “forest”) of decision trees which are each constructed using random subsets of the training data. Once a new point is passed into the RF model, each tree in the forest will classify that point and then RF will choose the class with the most “votes” in the forest. Each decision tree is made up of a collection of nodes that act like a path where each node is defined by a single feature with some threshold. The first node is the root node and connected to that are branches and then leaves. If we think of each of these nodes as possible stops on the tree’s path to a decision about a point , $x=(x^{1}, x^{2}, \ldots, x^{m})$, at each stop, the value of one of x’s entries will determine the next node it will stop at until it reaches a leaf node where the path ends and a decision is made based off of where it ended up. When growing each tree, a bootstrap sample of the training data is used and at each new node, a random subset of the variables is chosen, from which the best single predictor will be used to define the threshold that splits that node. This optimization of the individual decision tree is more of a greedy algorithm than an actual “optimization” problem in the classical sense. Consider an example where we are deciding how to best split a node with a random subset of $k\leq m$ features. For each feature $j$ in $\{1, \ldots, k\}$ choosing a threshold, $t$, will create two regions, one for the points where the value of the feature $j$ is less than or equal to $t$ and one where the value is greater than $t$ :

$$R_{1}=\left\{ {points x with x}_{j}\leq t \right\} and R_{2}=\left\{ {points x with x}_{j}>t \right\}$$

Then the feature $j$ and the threshold $t$ are chosen to be those that best classify the training data, i.e., those that minimize the classification error across the regions by satisfying:

$$\min_{j, t} \left[ \min_{} \sum_{x_{i}in R_{1}} {error}_{\left( j, s \right)}(x_{i})+\sum_{x_{i}in R_{2}} {error}_{\left( j, s \right)}(x_{i}) \right]$$

Where ${error}_{R\left( j, s \right)}(x)$ is the quantification of the error of classifying point $x_{i}$in region R using the variable j with threshold s. For classification, a popular metric to use here is the Gini Index. The end result is a robust model that is known to perform well in a wider variety of situations, but that is not very interpretable.

**Lasso regularization**

In this analysis, Lasso (Least Absolute Shrinkage and Selection Operator) regularization was used to perform dimensionality reduction in the first phase of the feature importance process. This was done to reduce noise and redundancy among the full set of variables; thus, the goal was to reduce the number of variables while still retaining the same amount of information. As seen in the results section of this paper, this goal was achieved.

When fitting a linear model to data, as mentioned above, there is an optimization problem being solved. In general, for a linear model of the form:

$$f\left( x,\beta\right)= \beta_{0}+\beta_{1}x^{1}+\ldots+ \beta_{m}x^{m}$$

You want to find the values of the coefficients, $\beta_{j}$, such that you are minimizing the loss function, $L(\beta)$. What Lasso regularization does is place a penalty on the size of the β coefficients where the optimization problem changes to finding the coefficients $\beta_{1}, \ldots, \beta_{m}$ such that we want to solve:

$$\min_{\beta} L\left( \beta\right)+ \lambda\sum_{j=1}^{m} \left| \beta_{j} \right|$$

Where λ > 0 [1]. Note that in our analysis, the values of the regularization parameters can be interpreted as $\frac{1}{\lambda} .$

The reason Lasso regularization was chosen for this analysis is that the penalty it places on the size of the β coefficients shrinks them in a way that forces some of the coefficients to 0 [1]. In doing this, the remaining features with non-zero coefficients can be interpreted as the subset of variables that were “chosen” by lasso regularization.

**Balanced accuracy for imbalanced data**

We used two metrics of model performance throughout the analysis: standard accuracy in the case of the aerobic threshold (5500 steps/day), which is the total proportion of correctly classified points in the test set:

$$\frac{true positives+true negatives}{true positives+true negatives+false positives+false negatives}$$

and balanced accuracy in the case of the home vs. community threshold (2500 steps/day), which is the arithmetic mean of the recall (or sensitivity) and specificity:

$$\frac{true positives}{true positive+false negatives}+ \frac{true negatives}{true negatives+false positives}$$

These metrics were chosen to ensure that the model performance metric used was representative of how well the model was performing on the target class.

The class distribution of each threshold was 58 (21.64%) home ambulators and 210 (78.36%) community ambulators in the case of the home vs. community threshold and 185 (69.03%) below the minimum aerobic activity threshold and 83 (30.97%) above for the aerobic threshold. While class distributions are not perfectly balanced in each case, the class imbalance in the case of the home vs. community threshold is starker at 1:4 and, more importantly, in favor of the negative class, unlike the aerobic threshold [2]. In the case of any significant class imbalance where the target class (the positive class) is the minority, the metric of standard accuracy could misrepresent how well the model is performing on the target class because standard accuracy may still be high, even if the model is not performing well on the target class [2]. In cases like this, we can use a metric like balanced accuracy, which allows the performance on the minority class to hold equal weight to that of the majority class and is also robust to misclassification noise [3].

**Results for the aerobic threshold (5500 steps/day) using balanced accuracy**

When using the metric of balanced accuracy for the aerobic threshold, the optimized regularization parameters were 2.9 for LR and 0.99 for linear SVM. Given that when we use standard accuracy, the optimal regularization parameters were very similar at 2.9 and 0.9, it follows that the results of the lasso regularization stage when using balanced accuracy were identical to the 16 features reported in the primary analysis: 6MWT, speed modulation, CCI age-adjusted score, PHQ-9, readiness to change stage score, ABC, usual orthotic and assistive device, marital status, years of education, gender, BMI, side of hemiparesis, time since initial stroke, number of medications, and ADI_N.

In the second stage, the drop column procedure was run using these 16 features. For LR, 10 features were found to be important, 6 of which were the important features found when using standard accuracy. In order of importance, these features were: readiness to change stage score, speed modulation, 6MWT, usual assistive device, number of medications, usual orthotic device, years of education, BMI, CCI age-adjusted score, and time since initial stroke. For SVM, the same four features were found to be important as when using standard accuracy. In order of importance, these features were: readiness to change stage score, number of medications, speed modulation, and 6MWT. Like SVM, RF also had four features found to be important, which, in order of importance were: speed modulation, usual assistive device, 6MWT, and PHQ-9. From these results, speed modulation was still found to be a primary characteristic for the aerobic threshold, in addition to 6MWT. BMI, CCI, PHQ-9, readiness to change stage score, time since initial stroke, number of medications taken, assistive and orthotic device use, and years of education were found to be ancillary characteristics. Supplemental Figure 1 displays the results for the drop column procedure for the aerobic threshold using balanced accuracy.

**S1 Fig. Drop column feature importance for aerobic threshold (5500 steps/day) using balanced accuracy.** Red markers show mean feature importance with 95% bootstrapped confidence interval. 6MWT and speed modulation were the only features found to be important across all three algorithms. *Abbreviations: ABC- Activities Specific Balance Confidence Scale, ADI_N- Area Deprivation Index (national percentile), BMI- body mass index, CCI- Charlson Comorbidity Index (age-adjusted), PHQ-9- Patient Health Questionnaire-9, Readiness_Stage- Readiness to change stage score, 6MWT- 6-Minute Walk Test, TSIS- time since initial stroke, LR- Logistic regression, SVM- Support vector machine, RF- Random forest.*

Importantly, these results are very similar to the results found when using standard accuracy. Note that when standard accuracy was used, 6MWT was found to be important for both SVM and LR but the 95% confidence interval for RF was only barely negative, resulting in 6MWT not being found to be a primary characteristic. It is encouraging that these results were found in this analysis because it is reflective of the fact that, even with mild class imbalance, standard accuracy was an appropriate choice of metric for this threshold.

**6-minute walk test as a single predictor of home vs. community thresholds (2500 steps/day)**

A precision-recall curve was used to assess the predictive ability of the 6MWT alone in predicting the home vs. community threshold (2500 steps/day; Supplemental Figure 2A). Similar to an ROC analysis, a precision-recall curve can be used to assess the skill of a prediction model, where:

$$Precision = \frac{True Positives}{(True Positives + False Positives)}$$

and

$$Recall= \frac{True Positives}{(True Positives + False Negatives)}$$

**S2 Fig. Precision-recall curve for predicting home vs. community ambulation using the 6-minute walk test (A) and aerobic threshold using the speed modulation (B).** The solid line plots the precision-recall curve, and the dashed line reflects a no-skill classifier (i.e., a model that cannot discriminate between classes). *Abbreviations: AUC- Area Under Curve.*

Recall is also referred to as sensitivity. As depicted in Supplemental Figure 2, a precision-recall curves plots the precision on the Y axis and recall on the X axis, where a point at (1,1) in the upper right corner of the figure would reflect a model with perfect precision and perfect recall. These curves can often be used instead of ROC curves in cases of class imbalance, as they are more sensitive to measures of recall. Similar to an ROC analysis, the area under the curve (AUC) can be computed to provide a measure of how well a model is performing where the AUC can have a maximum value of 1. A no-skill classifier (i.e., a model that randomly “guesses” the class) is depicted as a dashed line and changes based on the distribution of positive and negative cases:

*Precision (of a no-skill classifier) =* $\frac{Positive Cases}{(Positive Cases + Negative Cases)}$

Previous work suggests that a precision-recall plot is more informative than an ROC plot when evaluating a binary classifier in the presence of imbalanced data [4, 5], as was the case with the home vs. community threshold. For comparison purposes, we present the precision-recall curves for the 6-Minute Walk Test and speed modulation for predicting home vs. community and aerobic thresholds, respectively (S2 Fig).

As shown in Supplemental Figure 2A, the AUC for the 6MWT in predicting home vs. community ambulation is 0.642 which exceeds that of a no-skill classifier (precision of no-skill classifier = 58/(210 + 58) = 0.2164).

**Speed modulation as a single predictor of aerobic threshold (5500 steps/day)**

Supplemental Figure 2B displays the precision-recall curve for speed modulation in predicting the aerobic threshold of 5500 steps/day. The dashed line reflects a no-skill classifier and is calculated as: precision = 185/(83 + 185) = 0.6903. The AUC of 0.849 exceeds that of the no-skill classifier. When comparing the two precision-recall curves (and specifically the AUC values), it can be observed that speed modulation is a stronger predictor of the aerobic threshold than the 6MWT is in predicting the home vs. community threshold.

**Applying analysis with XGBoost**

It is worth noting that, for the two-phase drop column feature importance method used in this analysis, one need not use the three specified models applied here, as they were chosen on the basis of their performance on these problems in addition to their ease of application and prevalence of use. Depending on the amount of time and data one has access to, this method could be applied to a multitude of models, so long as the collection chosen can perform well on the given problem (i.e., minimally better than the uninformed model) and represent a collection of mathematically distinct algorithms.

Among the distinctions between the three models chosen in this analysis, perhaps the one that was made most apparent was that of the ensemble (RF) versus the non-ensemble (SVM and LR) in the case of the drop column results for the home vs. community threshold (see Fig 2). It would almost seem like the drop column results for RF in the home vs. community case are the opposite of the results for SVC and LR, save for 6MWT. As discussed in the discussion, we hypothesize that this could, at least partially, be contributed to the mathematical differences between how the RF model (the ensemble method) and the other models (the non-ensemble methods) are making decisions. One way we could test this hypothesis would be to run this analysis using another ensemble method which has been shown to perform well on clinical data, XGBoost.

Like RF, Extreme Gradient Boosting, or XGBoost (XGB), is a decision-tree-based ensemble method. XGB is distinct from RF, however, in that it is a “boosting” method that utilizes parallel computing (making it much faster), where RF is a “bagging” method, which is also known to take much longer to fit. The terms “bagging” and “boosting” refer to the methods by which points are resampled in the fitting process. Due to its efficient fitting times, we were able to run the analysis in two ways: first with a version of XGB that was tuned only for the parameter dictating the class weights (which is what was done in the original analysis) and second with a “tuned” XGB, where a more in-depth hyperparameter tuning was done to improve performance. For this analysis we used the *XGBoost* Python package [6].

For the minimally tuned XGB, the drop column results were nearly identical to those of RF in the main body of the paper with 6MWT being the only important feature for the home vs. community threshold and speed modulation being the only important feature for the aerobic threshold. This would support the supposition that the differences between the results of RF vs. LR and SVM, particularly in the home vs. community case, may be due in part to the mathematical distinctions between the ensemble and non-ensemble methods. With respect to model performance, for the home vs. community threshold, this minimally tuned XGB model had an average balanced accuracy of 66.5% (SD 5.4%, range 53.7% - 77.5%) with feature selection and 68.0% (SD 5.6%, range 54.9% - 84.5%) when using all features. For the aerobic threshold, it had an average accuracy score of 69.5% (SD 4.3%, range 59.2% - 80.2%) with feature selection and 69.7% (SD 4.6%, range 58.0% - 79.0%) when using all features.

For the tuned version, we used GridSearchCV from sklearn to tune the parameters *min_child_weight*, *max_depth*, and *scale_pos_weight* (which is the XGB equivalent to the *class_weight* parameter tuned in the the original analysis). After again running the drop column procedure using this tuned XGB, we found that 6MWT as well as ADI_N were found to be important for the home vs. community threshold; for the aerobic threshold, 6MWT and speed modulation were found to be important. Again, note that these results are much more closely aligned with those given by RF in the original analysis, particularly for the aerobic threshold. As for performance, in the case of the home vs. community threshold, the tuned XGB had an average balanced accuracy score of 73.7% (SD 4.9%, range 61.4% - 86.1%) with feature selection and 72.9% (SD 5.3%, range 60.7% - 84.6%) when using all features. For the aerobic threshold, it had an average accuracy score of 72.5% (SD 4.0%, range 57.6% - 79.0%) with feature selection and 72.3% (SD 4.2%, range 57.6% - 81.5%) when using all features.

Additionally, it is important to note that the results of this additional analysis further support the idea that 6MWT and speed modulation are fundamentally important features to the home vs. community and aerobic threshold classification problems, respectively, as they are still the prevailing features when considering the intersection of important features across algorithms.

We refer the reader to the reference list below for further reading on these topics.

**References**

1. Friedman J, Hastie T, Tibshirani R. The elements of statistical learning: Springer series in statistics New York; 2001.

2. Krawczyk B. Learning from imbalanced data: open challenges and future directions. Progress in Artificial Intelligence. 2016;5(4):221-32. doi: 10.1007/s13748-016-0094-0.

3. Ferri C, Hernández-Orallo J, Modroiu R. An experimental comparison of performance measures for classification. Pattern Recognition Letters. 2009;30(1):27-38. doi: 10.1016/j.patrec.2008.08.010.

4. He H, Garcia EA. Learning from Imbalanced Data. IEEE Transactions on Knowledge and Data Engineering. 2009;21(9):1263-84. doi: 10.1109/TKDE.2008.239.

5. Saito T, Rehmsmeier M. The Precision-Recall Plot Is More Informative than the ROC Plot When Evaluating Binary Classifiers on Imbalanced Datasets. PloS one. 2015;10(3):e0118432. doi: 10.1371/journal.pone.0118432.

6. *XGBoost documentation* XGBoost Documentation- xgboost 1.5.2 documentation. (n.d.) [April 13, 2022]. Available from: <https://xgboost.readthedocs.io/en/stable/>
